# Supplementary material for: Food insecurity status and determinants among Urban Productive Safety Net Program beneficiary households in Addis Ababa, Ethiopia
Source: PLoS One. 2021 Sep 27;16(9):e0256634. doi: 10.1371/journal.pone.0256634 (PMC8476016; doi:10.1371/journal.pone.0256634)
Supplement: S1 File — (DOCX) [file pone.0256634.s001.docx]

**English version Questionnaire**

Participant ID No___________ Woreda ____ Interviewer name_________________________ Date of interview____________ Starting time _____________Ending time___________

Please encircle the correct answer which to indicate number and write a correct number on the space provided.

| **Part I: Socio-demographic and economic characteristics** | | | |
| --- | --- | --- | --- |
| **S.No** | **Questions** | **Answer Option** | **Skip** |
| Q101 | Sex of household head | 1. Female 2. Male |  |
| Q102 | Age of household head in year | _______________ |  |
| Q103 | Marital status of the household head | 1. Unmarried 2. Divorced 3. Widowed 4. Married/live together |  |
| Q104 | Educational status of Household head | 1. No education  2. Primary level education  3. The secondary level of education  4. Above the secondary level |  |
| Q105 | Occupation status of Household head | 1. Safety network 2. Daily labor/informal activities 3. Pension/ labor poor 4. No work 5. Private work _________ |  |
| Q106 | Total number of family size | _______________ |  |
| Q107 | Number of individual under 18 years old | _______________ |  |
| Q108 | Labor force member in the household(18-64 years) | _______________ |  |
| Q 109 | History of any chronic medical problem confirmed by the physician. | 1. Yes 2. No |  |
| Q 110 | Source of household food | 1. Prepared at home 2. Buying at restaurant 3. Other (specify)_________ |  |
| Q 111 | Food purchasing responsible gender | 1. Male 2. Female |  |
| Q 112 | Food expenditure from monthly income of household (in ETB) | _____________ |  |
| Q 113 | Ownership of the house | 1. Rental from private 2. Rental from government 3. Private 4. Other specify(_______) |  |
| Q 114 | Monthly payment from UPSNP(in ETB) | _____________ |  |
| Q 115 | Number of months paid from safety net program | _____________ |  |
| Q 116 | Additional source of income of safety net beneficiary manly | 1. From Safety net only 2. From Daily laborer 3. From Pension 4. From Private work |  |
| Q 117 | Total monthly income of household from all source (in ETB) | ______________ |  |
| Q 118 | Access to credit service | 1. No 2. Yes |  |
| Q 119 | Access to free health service | 1. No 2. Yes |  |

| **Part II: Household Food Insecurity Access Scale (HFIAS) Questions** | | | |
| --- | --- | --- | --- |
| **S.No** | **Question** | **Option** | **Skip** |
| Q201 | In the past four weeks, did you worry that your household would not have enough food? | 1. Yes 2. No | Q202 |
| Q201a | How often did this happen? | 1. Rarely 2. Sometimes 3. Often |  |
| Q202 | In the past four weeks, were you or any household member not able to eat the kinds of foods you preferred because of a lack of resources? | 1. Yes 2. No | Q203 |
| Q202a | How often did this happen? | 1. Rarely 2. Sometimes 3. Often |  |
| Q203 | In the past four weeks, did you or any household member have to eat a limited variety of foods due to a lack of resources? | 1. Yes 2. No | Q204 |
| Q203a | How often did this happen? | 1. Rarely 2. Sometimes 3. Often |  |
| Q204 | In the past four weeks, did you or any household member have to eat some foods that you did not want to eat because of a lack of resources to obtain other types of food? | 1. Yes 2. No | Q205 |
| Q204a | How often did this happen? | 1. Rarely 2. Sometimes 3. Often |  |
| Q205 | In the past four weeks, did you or any household member have to eat a smaller meal than you felt you needed because there was not enough food? | 1. Yes 2. No | Q206 |
| Q205a | How often did this happen? | 1. Rarely 2. Sometimes 3. Often |  |
| Q206 | In the past four weeks, did you or any household member have to eat fewer meals in a day because there was not enough food? | 1. Yes 2. No | Q207 |
| Q206a | How often did this happen? | 1. Rarely 2. Sometimes 3. Often |  |
| Q207 | In the past four weeks, was there ever no food to eat of any kind in your household because of a lack of resources to get food? | 1. Yes 2. No | Q208 |
| Q207a | How often did this happen? | 1. Rarely 2. Sometimes 3. Often |  |
| Q208 | In the past four weeks, did you or any household member go to sleep at night hungry because there was not enough food? | 1. Yes 2. No | Q209 |
| Q208a | How often did this happen? | 1. Rarely 2. Sometimes 3. Often |  |
| Q209 | In the past four weeks, did you or any household member go a whole day and night without eating anything because there was not enough food? | 1. Yes 2. No | Q209a |
| Q209a | How often did this happen? | 1. Rarely 2. Sometimes 3. Often |  |

**Thank you for your participation and valuable information.**
