# Supplementary material for: Food insecurity status and determinants among Urban Productive Safety Net Program beneficiary households in Addis Ababa, Ethiopia
Source: PLoS One. 2021 Sep 27;16(9):e0256634. doi: 10.1371/journal.pone.0256634 (PMC8476016; doi:10.1371/journal.pone.0256634)
Supplement: S2 File — (DOCX) [file pone.0256634.s002.docx]

**ለጥናቱ ተሳታፊዎች የሚሰጥ መረጃ (Amharic version of information sheet)**

እንደምን አረፈዳችሁ/ዋላቹህ፡-ስሜ-----------------------ይባላል፡፡ የመጣሁት የጥናቱ ተመራማሪ የሆነዉን አትመን ደርሶን ወክየ መረጃ ለመሰብሰብ ነዉ፡፡

**የጥናቱ ርዕስ፡-** በአዲስ አበባ ከተማ ስር በሚኖሩ የምግብ ዋስትና ተጠቃሚዎች ላይ የምግብ እጥረት ስርጭት እና ተያያዥ ወሳኝ መንስኤዎች መለየት ነው፡፡

1. የጥናቱ ተመራማሪ ሙለ ስም፡- አትመን ደርሶ

2. የመረጃ ሰብሳቢ ተቆጣጣሪ ሙለ ስም፡ --------------------

**የጥናቱ አላማ፡** የጥናቱ አላማ በአዲስ አበባ ከተማ ስር በሚኖሩ የምግብ ዋስትና ተጠቃሚዎች ላይ የምግብ እጥረት ስርጭት እና ተያያዥ ወሳኝ መንስኤዎች መለየት ነው፡፡ ጥናቱ የሚሰራው ለ2ኛ ድግሪ የስነምግብ ማህበረሰብ ጤና ለመመረቂያ ጹሁፍ ጥናት ማሟያ ነው፡፡

**የጥናቱ አካሄድ እና ተሳትፎ፡** የጥናቱ መስሪያ መንገድ ከአጭር ግዜ መረጃ በመሰብሰብ የሚካሄድ ሲሆን የጥናቱ ተሳታፉ በአንድ ጊዜ ከመረጃ ሰብሳቢው ጋር ከ 20 ደቂቃ በላይ የጊዜ ቆይታ አይወስድም፡፡ ለጥናቱ የእርስዎ ተሳታፎ የሚያስፈሌገው የሚሰጡት መረጃ ታማኝነት ስላለውና ጥናቱን ሰርቶ ለማጠናቀቅ በጣም አስፈላጊ ስለሆኑ ነው፡፡ ነገር ግን እርስዎ እንዲሳተፉ የተመረጡት አሳታፉ በሆኑ አካሄድ ነዉ::

**ምስጢራዊነት፡** የሚሰጡኝ መረጃ በደንብ የሚያዝ ሲሆን ለማንም ሰው ተላልፎ አይሰጥም፡፡ ከዚህ በተጨማሪም ከመረጃው ጋር የእርስዎ ስም አይያያዝም፡፡ ስለዚህም መረጃ ሰብሳቢው የሚጠቀመው ኮድ ነው፡፡ የሰጡት መረጃም ተቆልፎ በመረጃ መያዣ ነው የሚቀመጠው፡፡ስለሆነም ከጥናቱ ተመራማሪና ከመረጃ ሰብሳቢዎች ተቆጣጣሪ ውጭ ማንም አያገኘውም፡፡የጥናቱ ተመራማሪም መረጃውን ከሚፈለገዉ አላማ ዉጭ አይጠቀምበትም፡፡ ጥናቱ ተሰርቶ አልቆ የጥናቱ ተመራማሪ ከተመረቀ በኃላ መረጃ የተሞላበት መጠይቅ በጥንቃቄ ይቃጠላል፡፡

**ጥቅም፡** ጥናቱ ለአጭር ጊዜ የሚሆን የገንዘብ የጤና እና የአቅም ግንባታ ድጋፍ አያደርግም፡፡ ነገር ግን ከተወሰነ ጊዜ በኃላ የጥናቱ ዉጤት ለሚመለከታቸው መስሪያ ቤቶች፤ ለህግ አርቃቂዎች፡ለእቅድ አውጭዎች የሚያገለግል ሲሆነ ይህም ለከተማ ውስጥ ኗሪዎች የምግብ እጥረት ለመከላከል ትልቅ አስተዋፅኦ ያደርጋል፡፡

**ጉዳት፡-** ጥናቱ ምንም አይነት አካላዊ፤ እምሮአዊ፤ ማህበራዊ እና ኢኮኖሚያዊ ጉዳት አያስከትልም፡፡

**ጥቅማጥቅምና ማካካሻ:** ጥናቱ ምንም አይነት ጥቅማጥቅም እና ማካካሻ አይኖረዉም ምክንያቱም ጥናቱ ምንም አይነት ጉዳት ስለማያስከትል፡፡

**ግንኙነትን በተመለከተ፡** መረጃ ሰጪው ተጨማሪ መረጃ ማግኘት ከፈለገ በማንኝውም ጊዜ መጠየቅ ይችላል፡፡ስለዚህም የጥናቱ ተመራማሪን ወይም የመረጃ ሰብሳቢ ተቆጣጣሪዎችን ማነጋገር ይችላል፡፡ ከዚህ በተጨማሪም ጥናቱ የሚሰራው ቅዱስ ጳዉሎስ ሆስፒታል ሚሊንየም ሜዲካል ኮሌጅ በሰጠው ፈቃድ ስለሆነ መጠየቅ ከፈለጉ የሚከተለውን አድራሻ ይጠቀሙ፡፡

**የማቋረጥ ነፃነት፡** መረጃ ሰጭዎች መጠይቁ በሚካሄድበት ጊዜ ደስተኛ ካልሆኑ መረጃ መስጠቱን ማቋረጥ ይችላሉ፡፡ ሲያቋርጡም ምንም አይነት ተፅእኖ አይደርስበዎትም፡፡

1. ቅዱስ ጳዉሎስ ሆስፒታል ሚሊንየም ሜዲካል ኮሌጅ ቢሮ

ስልክ ቁጥር፤**+251 (0) 112732639**

1. የጥናቱ ተመራማሪ ሙሉ ስም እና አድራሻ፣ አትመን ደርሶ

ስልክ ቁጥር፡ +251 (0) 940286156፤ E-mail: embule2015@gmail.com

1. የመረጃ ሰብሳቢ ተቆጣጣሪ ሙለ ስም እና አድራሻ __________________

**የስምምነት መግለጫ ቅፅ(amharic version of consent form)**

**የጥናቱ ርዕስ፡-** በአዲስ አበባ ከተማ ስር በሚኖሩ የምግብ ዋስትና ተጠቃሚዎች ላይ የምግብ እጥረት ስርጭት እና ተያያዥ ወሳኝ መንስኤዎች መለየት ነው፡፡

አቶ አትመን ደርሶ የጥናቱ ተመራማሪ ባለቤት እደሆነ አውቃለሁ፡፡ የጥናቱም አላማ በሚገባኝ ቋንቋ የተገለጸልኝ ሲሆን አላማውም በአዲስ አበባ ከተማ በሚኖሩ የምግብ ዋስትና ተጠቃሚዎች ላይ የምግብ እጥረት ስርጭት እና ተያያዥ ወሳኝ መንስኤዎች ማጥናት ነው፡፡ እኔ የምሰጠው ሃሳብም በጥንቃቄ እንደሚያዝና ለሌላ ሰው ተላልፎ እንደማይሰጥ ተነግሮኛል፡፡ ጥናቱ ለኔም ሆነ ለቤተሰቤ ምንም አይነት ጉዳት እንደማያደርስና ምንም የአጭር ጊዜ ጥቅምም ሆነ ማካካሻ እንደማይሰጠኝ አምኛለሁ፡፡ከዚህ በተረፈም የምሰጠው ሃሳብም በወረዳዬ የማገኘውን ጥቅም ወይም ክፍያ እንደማይቀንስብኝ ተነግሮኛል፡፡ስለዚህ ጥናቱ ያልገባኝን ነገር መረጃው ከመሰብሰቡ በፊትም ሆነ በሌላ ጊዜ ከዚህ በታች የተጠቀሱትን አድራሻ በመጠቀም መጠየቅ እንደምችል ተነግሮኛል፡፡ከዚህም በላይ ያለዉን ቅፅ አንብቢያለዉ ወይም በሚገባኝ ቋንቋ ተነቦልኝ የምጠየቀዉን ጥያቄ ለመመለስ ፈቃደኛ ሆኜ ተስማምቻለሁ፡፡

አዎ ፈቃደኛ ነኝ ፉርማ__________ ፈቃደኛ አይደለሁም

(ለመሳተፍ ፍቃደኛ ካልሆኑ አመስግናለዉ ይለፉ)

የመረጃ ሰብሳቢ ስም _________________ፉርማ_______ ቀን________

1. ቅዱስ ጳዉሎስ ሆሰፒታል ሚሊንየም ሜዲካል ኮሌጅ ቢሮ

ስልክ ቁጥር፤ **+251 (0) 112732639**

1. የጥናቱ ተመራማሪ ሙሉ ስም እና አድራሻ አትመን ደርሶ ፤

ስሌክ ቁጥር፡ +251 (0) 940283156፣ E-mail: embule2015@gmail.com

1. የመረጃ ሰብሳቢ ተቆጣጣሪ ሙለ ስም እና አድራሻ __________________

**አማርኛ መጠይቅ(Amharic questionnaire)**

የተሳታፊ መለያ ቁጥር(**ID**)______ወረዳ ____የቃል መጠይቅ አድራጊው ስም_________ ቀን_______________ የተጀመረበት ሰዓት________የተጠናቀቀበት ሰዓት________

ጥያቄዉን በትክክል በማንበብ ተጠያቂዉ የሚሰጠዉን መልስ በትክክል አክብብ፡፡ በስድ የሚመለሱ ጥያቆዎችን በተቀመጠዉ ክፍት ቦታ ላይ ጻፍ፡፡

| **ክፍሌ 1፡ የሶሽዮ-ዲሞግራፊና ኢኮኖሚካል መረጃ መጠይቅ** | | | |
| --- | --- | --- | --- |
| ተ.ቁ | ጥያቄ | አማራጭ | እለፍ |
| 101 | የቤቱ አስተዳዳሪ ጾታ | 1. ሴት 2. ወንድ |  |
| 102 | የቤቱ አስተዳዳሪ እድሜ (በአመት) | _______________ |  |
| 103 | የቤቱ አስተዳዳሪ የጋብቻ ሁኔታ | 1. ያላገባ/ች 2. የፈታ/ች 3. የሞተበት/የሞተባት 4. ያገባ/ች |  |
| 104 | የቤቱ አስተዳዳሪ የትምህርት ሁኔታ | 1. ማንበብና መፃፍ የማትችል/የማይችል 2. አንደኛ ደረጃ 3. ሁለተኛ ደረጃ 4. ከሁለተኛ ደረጃ በላይ |  |
| 105 | የቤቱ አስተዳዳሪ የስራ ሁኔታ | 1. ሴፍቲኔት ስራ 2. የቀን ስራተኛ 3. ጡረተኛ/አቅመደካማ 4. ስራ የሌለዉ/የሌላት 5. ሌላ ካለ ይግለጹ___ |  |
| 106 | በቤት ዉስጥ ያሉ የቤተሰብ ብዛት | _______________ |  |
| 107 | ከ18 አመት በታች የቤተሰብ አባል ብዛት | _______________ |  |
| 108 | እድሚያቸዉ ከ18-64 አመት የሆኑ የቤተሰብ አባላት ብዛት | _______________ |  |
| 109 | በሃኪም የተረጋገጠ የቀደመ የጤና ችግር አለብዎ? | 1. አዎ 2. የለም |  |
| 110 | ቤተሰቡ በዋናነት ምግብ ከየት ነዉ የሚያገኘዉ? | 1. ቤት ዉስጥ በማዘጋጀት 2. ከገብያ በመግዛት 3. ሌላ (ካለ ይግለጹ)_______ |  |
| 111 | ከቤተሰቡ መካከል በዋናነት ምግብ ከገበያ የመግዛት ኃላፊነት የማነዉ? | 1. ወንድ 2. ሴት |  |
| 112 | አማካኝ ወርሀዊ የምግብ ወጭ ምን ያህል ነዉ(በኢትጵያ ብር)? | _____________ |  |
| 113 | የምትኖሩበት ቤት ባለቤቱ ማን ነዉ? | 1. ከግለሰብ ተከራይተዉ 2. ከመንግሰት ተከራይተዉ 3. የግልዎ ቤት |  |
| 114 | በየወሩ ከምግብ ዋስትና ጽፈት ቤት የሚያገኙት ብር መጠን (በኢትዮጲ ብር)? | _____________ |  |
| 115 | የምግብ ዋስትና ፕሮግራመ መጠቀም ከጀመሩ ምንያክል ወር ሆነዎት? | _____________ |  |
| 116 | ተጨማሪ ወርሃዊ የቤት ገቢ ምንጭ ከምንድን ነዉ? | 1. ከሴፍቲኔት ብቻ 2. ከቀን ስራ 3. ከጡረታ 4. ከግል ስራ |  |
| 117 | አማካኝ ወርሃዊ የቤት ገቢ ምንያክል ነዉ (በኢትዮጲያ ብር)? | ______________ |  |
| 118 | የብድር አገልግሎት ተጠቃሚ ነዎት? | 1. የለም 2. አዎ |  |
| 119 | የነጻ ህክምና አገልግሎት ተጠቃሚ ነዎት? | 1. የለም 2. አዎ |  |

| **ክፍል 2: የቤተሰብ የምግብ ዋስትናን በተመለከተ መጠይቅ** | | | |
| --- | --- | --- | --- |
| **ተ.ቁ** | **ጥያቄ** | **አማራጭ** | **ዝለል** |
| 201 | ባአለፈዉ 4 ሳምንት በቤተሰብህ/ሽ ዉስጥ ስለ ምግብ ማለቅ ተጨንቀሽ/ህ ታውቂያለሽ/ታዉቃለህ? | 1. አዎ 2. የልም | 202 |
| 201ሀ | ተጨንቀዉ ካወቁ ለምን ያህል ጊዜ? | 1. ከ1-2 ጊዜ 2. ከ3-10 ጊዜ 3. ከ10 ጊዜበላይ |  |
| 202 | ባአለፈው 4 ሳምንታት ውስጥ በምግብ ማለቅ ወይም ምግብ መግዣ የሚሆን ገንዘብ በማጣትዎ ምክንያት የቤተሰብዎ አባል ወይም እርሰዎ የፈለጉትን/የመረጡትን ምግብ ሳይመገቡ ቀርተዉ ያዉቃሉ? | 1. አዎ 2. የለም | 203 |
| 202ሀ | ሳይመገቡ ቀርተዉ ከወቁ ለምንክል ጊዜ? | 1. ከ1-2 ጊዜ 2. ከ3-10 ጊዜ 3. ከ10 ጊዜበላይ |  |
| 203 | ባአለፈው 4 ሳምንታት ውስጥ በምግብ ማለቅ ወይም ምግብ መግዣ የሚሆን ገንዘብ በማጣትዎ ምክንያት የቤተሰብዎ አባል ወይም እርሰዎ ከተለያዩ የምግብ ዝርዝሮች በመቀነስ ተመግበዉ ያዉቃሉ? | 1. አዎ 2. የለም | 204 |
| 203ሀ | ቀንሰዉ ተመግበዉ የሚያዉቁ ከሆነ ለምን ያህል ጊዜ? | 1. ከ1-2 ጊዜ 2. ከ3-10 ጊዜ 3. ከ10 ጊዜበላይ |  |
| 204 | ባአለፈው 4 ሳምንታት ውስጥ በምግብ ማለቅ ወይም ምግብ መግዣ የሚሆን ገንዘብ በማጣትዎ ምክንያት የቤተሰብዎ አባል ወይም እርሰዎ መብላት ከማይፈልጉት የምግብ አይነት ዉስጥ ተመግበዉ ያዉቃሉ? | 1. አዎ 2. የለም | 205 |
| 204ሀ | ተመግበዉ የሚያዉቁ ከሆነ ለምን ያህል ጊዜ? | 1. ከ1-2 ጊዜ 2. ከ3-10 ጊዜ 3. ከ10 ጊዜ በላይ |  |
| 205 | ባአለፈው 4 ሳምንታት ውስጥ በቂ ምግብ ባለመኖሩ ምክንያት የቤተሰብዎ አባል ወይም እርሰዎ የምትመገቡትን ምግብ መጠን ቀንሰዉ ተመግበዉ ያወቃሉ? | 1. አዎ 2. የለም | 206 |
| 205ሀ | የምግብ መጠን ቀንሰዉ ተመግበዉ ካወቁ ለምን ያህል ጊዜ? | 1. ከ1-2 ጊዜ 2. ከ3-10 ጊዜ 3. ከ10 ጊዜ በላይ |  |
| 206 | ባአለፈው 4 ሳምንታት ውስጥ በቂ ምግብ ባለመኖሩ ምክንያት የቤተሰብዎ አባል ወይም እርሰዎ በቀን ዉስጥ የምትመገቡበት ምግብ ጊዜ ዘለዉ ያወቃሉን? | 1. አዎ 2. የለም | 207 |
| 206ሀ | የምግብ ጊዜን ዘለዉ የሚያቁ ከሆነ ለምንያህል ጊዜ? | 1. ከ1-2 ጊዜ 2. ከ3-10 ጊዜ 3. ከ10 ጊዜ በላይ |  |
| 207 | ባአለፈው 4 ሳምንታት ውስጥ ምግብ መግዣ የሚሆን ገንዘብ በማጣትዎ ምክንያት በቤትዎ ዉስጥ ምንም አይነት የሚበላ ምግብ አጥተዉ ያዉቃሉ? | 1. አዎ 2. የለም | 208 |
| 207ሀ | ምንም አይነት የሚበላ ምግብ ካልነበረ ለምን ያህል ጊዜ? | 1. ከ1-2 ጊዜ 2. ከ3-10 ጊዜ 3. ከ10 ጊዜ በላይ |  |
| 208 | ባአለፈው 4 ሳምንታት ውስጥ በቂ ምግብ ባለመኖሩ ምክንያት የቤተሰብዎ አባል ወይም እርሰዎ በማታ እየራበዎት ወደምኝታ ሄደዉ ያዉቃሉ? | 1. አዎ 2. የለም | 209 |
| 208ሀ | እየራበዎት ወደምኝታ ሄደዉ ካወቁ ለምንያህል ጊዜ? | 1. ከ1-2 ጊዜ 2. ከ3-10 ጊዜ 3. ከ10 ጊዜ በላይ |  |
| 209 | ባአለፈው 4 ሳምንታት ውስጥ በቂ ምግብ ባለመኖሩ ምክንያት የቤተሰብዎ አባል ወይም እርሰዎ እየራበዎት ለ 24 ስዓት ምንመአይነት ምግብ ሳይበሉ ቀርተዉ ያዉቃሉ? | 1. አዎ 2. የለም |  |
| 209ሀ | ለ 24 ስዓት ምንመአይነት ምግብ ሳይበሉ ቀርተዉ ካወቁ ለምንያህል ጊዜ? | 1. ከ1-2 ጊዜ 2. ከ3-10 ጊዜ 3. ከ10 ጊዜበላይ |  |

ስለተሳትፎዎና ስለሰጡን መረጃ በጣም አመስግናለሁ.
