# Supplementary material for: Food insecurity status and determinants among Urban Productive Safety Net Program beneficiary households in Addis Ababa, Ethiopia
Source: PLoS One. 2021 Sep 27;16(9):e0256634. doi: 10.1371/journal.pone.0256634 (PMC8476016; doi:10.1371/journal.pone.0256634)
Supplement: Attachment — Submitted filename: ccb_Manusccript Review_Food Insecurity Status and Determinants among Urban Productive Safety Net Program Beneficiary Households in Ethiopia.docx [file pone.0256634.s004.docx]

**Food Insecurity Status and Determinants among Urban Productive Safety Net Program Beneficiary Households in Ethiopia, Manuscript PONE-D-20-30124**

Atimen Derso^1^, Hailemichael Bizuneh^2^, Awoke Keleb^1^, Metadel Adane^1*^

^1^Department of Environmental Health, College of Medicine and Health Sciences, Wollo University, Dessie, Ethiopia.

^2^Department of Public Health, Saint Paul’s Hospital Millennium Medical College, Addis Ababa, Ethiopia

*******[**metadel.adane2@gmail.com**](mailto:metadel.adane2@gmail.com)

**Abstract**

**Background**

Measuring household food insecurity in specific geographic areas is vital for providing information that enables effective intervention measures to be taken. Therefore, this study aimed to assess the prevalence of food insecurity and associated factors among Urban Productive Safety Net Program (UPSNP) beneficiary households in Addis Ababa, Ethiopia’s capital city.

**Methods**

A community based cross-sectional study was conducted among 624 UPSNP beneficiary households from June to July, 2019. A multi-stage sampling method was used and study participants were selected using simple random sampling technique after proportionally allocated sample size for 9 districts. Data were collected by trained personnel using a pretested structured questionnaire. The outcome variable food insecurity was measured by Household Food Insecurity Access Scale (HFIAS), a tool that has been developed by Food and Nutrition Technical scale (FANTA) and validated for developing countries, including Ethiopia. Multivariable logistic regression was used to identify independent predictors of household food insecurity. By adjusting the confounders, variable at *p*-value < 0.05 at 95% CI (confidence interval) was declared significantly associated with household food insecurity.

**Results**

The prevalence of household food insecurity was 77.1% [95%CI:73.8-80.7%] during the month prior to the survey. From the multivariable analysis, illiteracy of household head [AOR: 2.56; 95%CI:1.08-6.07], family size of 4 or above [AOR: 1.87; 95%CI:1.08-3.23]; high dependency ratio [AOR: 3.95; 95%CI:1.31-11.90]; household lacking access to credit [AOR:2.85; 95%CI:1.25-6.49] and low household income [AOR: 4.72; 95%CI:2.32-9.60] and medium household income [AOR: 9.78; 95%CI:4.29-22.35] were significantly associated with household food insecurity.

**Conclusion**

We found that three in four of Addis Ababa’s UPSNP beneficiary households had a food insecurity problem. Implementation of measures to improve household income, minimize dependency ratio of households, and arrange access to credit services are very important to tackle food insecurity problems.

**Keywords:** Food insecurity, urban productive safety net program, Addis Ababa, Ethiopia

**Introduction**

Household food insecurity exists when a family does not have adequate physical, social and economic access to sufficient, safe and nutritious food to meet the dietary needs and food preferences of its members for an active and healthy life [1, 2]. It is one of the underlying causes of all forms of malnutrition, including the quality, quantity, and continuity of diet [3, 4] that continues to be the major challenge around the world.

Lack of access to sufficient and nutritious food is a significant challenge to achieving international nutritional targets with respect to children. For instance, globally, only half (51%) of children aged 6 to 23 months get the recommended minimum number of daily meals, and only 16% of them eat a minimally acceptable diet [5].

The prevalence of poverty and the urban population in Africa are both growing rapidly[6], and these numbers are increasing the challenge of meeting the food security need of the urban population [7]. Members of this poor urban population experience under-nutrition, micronutrient deficiencies, and overweight and obesity at the same time. Currently, one in three undernourished children live in an urban setting, a proportion that will increase over the next decade in the response to globalization, migration, population growth, income inequality and climate change across low- and middle-income countries [8-10].

Food insecurity is a major public health problem that appears to be increasing in almost all regions of Africa, causing about 52.5% of population to be exposed to moderate or severe food insecurity. In Sub-Sahara and Eastern Africa, for instance, the magnitude of food insecurity among their populations in 2018 was 57.7% and 62.7%, respectively. In Eastern Africa, as a consequence of food insecurity more than one-third of populations (30.8%) were undernourished due to a lack of sufficient calories [3].

Food insecurity is manifested in the reduction of food consumption and lack of access to food variety and diversity [11, 12] that affects per capita food consumption. The inadequate access to food (both quality and quantity) causes inequality of food distribution among family members, especially to infants and children [13] as well as anxiety and stress among women in the household [14]. Ultimately, a lack of food access affects the physical and mental development of an individual and the economic productivity of a population and a country [3, 15].

Action is needed as the severity of food insecurity in Ethiopia has gotten worse in recent years; 8.5 million people lack food security as a result of population displacement, political instability, armed clashes, persistent drought and increase food prices [16]. Food insecurity has an indirect significant contribution to under nutrition that costs 16.5% of gross domestic product (GDP), 28% of under-five mortality, 15.8% of students repeating a grade, and nearly 13 billion Ethiopian birr lost in productivity in manual activities [17]. In Ethiopia, only 45% of children aged 6 to 23 months get the recommended minimum number of meals, and only 7% of them eat a minimally acceptable diet [18].

In Ethiopia, the Urban Productive Safety Net Program (UPSNP) aims to improve nutritional status and address the underlying causes of food insecurity by providing beneficiaries food and cash in rural and urban areas [15]. However, the program is new in urban areas, launched in Ethiopia in 2017 with Addis Ababa as one of the pilot areas. Great efforts to improve food security in Ethiopia have been made, but the plausibly of urban UPSNP contributing to a lower prevalence of household food insecurity in urban settings including Addis Ababa and related predictors have not been investigated yet.

Considering this context and area-specific knowledge gap, this study aims to assess the prevalence of food insecurity and associated factors among UPSNP beneficiary households in Ethiopia’s capital city, Addis Ababa. One of the goals of this study is to contribute to the scaling up the urban safety net program, thereby helping to change the food security status of Addis Ababa’s urban poor.

**Methods and Materials**

## Study Design and Study Area

A community-based cross-sectional study was conducted from June to July, 2019. The study was conducted in the capital city of Ethiopia, Addis Ababa. Addis is also the headquarters of the African Union. This study covered 9 districts (called *woredas* in Amharic, the local language) found in three of Addis Ababa’s ten sub-cities (Arada, Lideta and Yeka sub-cities). However, the Urban Safety Net Program was implemented in 90 selected districts (of 116 existing districts) across all 10 sub-cities. About 78,543 households have been participating in UPSNP since 2017 [19]. According to the projection of the 2007 Ethiopian population census, the total population of the city in 2017 was 4,567,857 with an estimated 913,572 households [20].

The national nutrition policy of Ethiopia addresses food insecurity through preventive approaches via nutrition-sensitive interventions that address underlying causes such as poverty [21]. The government of Ethiopia, World Food Program (WFP) and Development Partners have worked together to improve food security and stabilizing assets covering 10 million people in the country through the UPSNP [19].

The UPSNP targets urban poorer households (earning below $2 per day) with nutritionally vulnerable members in the identified pilot cities including Addis Ababa and some regional towns with interventions that improve their living and food security levels [19].

**Study Population, Inclusion and Exclusion Criteria**

All UPSNP beneficiary households in Addis Ababa were the source populations of the study. The study population was all UPSNP beneficiary households in the systematically selected districts included in this study*.* From the study population, respondents in the households who were unable to communicate for different reasons such as illness or mental problems were excluded. Registered and active UPSNP beneficiary households in the selected districts at the time of data collection were included.

## Sample Size Determination and Sampling Procedure

Sample size was determined using a single proportion formula [$n= \frac{{[ (z}^{2}\frac{\alpha}{2})*(p\left( 1-p \right)]}{w^{2}}$ ] with the assumption of the prevalence (p) of household food insecurity was 58.16%, taken from a previous study in Addis Ababa [22], 95% confidence interval (CI) (Z*a*/2 = 1.96) and *w* marginal error (5%). A 1.5 design effect was also considered, and then 10% contingency for non-response rate included. Finally, we obtained a final sample size of 624 households.

We used a multistage sampling of three-stages. During the first stage, out of Addis Ababa’s 10 sub-cities, 30% were selected (the 3 sub-cities Arada, Lideta and Yeka) by probability proportional to size sampling. Then, in the second stage, from selected sub-cities, 30% of the 30 active districts implementing the program were again selected by probability proportional to size sampling, which is 9 districts; and then sample size was proportionally allocated to the 9 districts. Finally, using their payment code list already available in the respective districts, the third stage of sampling unit (household) was selected using simple random sampling method. From the selected household, the study participants were selected based on their experience of household food status according to FANTA guidelines [23].

## Variable measurement

## Outcome variable

This study outcome variable was household food insecurity status [“insecure household = 1” or “secure household = 0”] during the month previous to the survey. We used a standard tool of Household Food Insecurity Access Scale (HFIAS), a tool that has been developed by Food and Nutrition Technical Assistance (FANTA) and validated for developing countries, including Ethiopia. HFIAS has three domains: anxiety and uncertainty domain, insufficient quality domain, and insufficient food intake (quantity) and its physical consequences domain [23].

HFIAS has a set of nine closed-ended questions categorized as “yes or no”, which represent occurrence; and under each “yes” category 3 option of occurrence frequency [rarely = 1, sometimes = 2, and often = 3].

The status of household food insecurity was estimated into two categories [food insecure and food secure] using prevalence of insecure household food access. Then, household food insecurity status was classified as mild, moderate or severe based on the frequency of the occurrence of each item in Table 1.

**The presence of food secure households** was declared when household score [item one = ‘0’ or ‘1’ and items two to nine = ‘0’].

**The presence of food insecure households** was declared when household score [item one = ‘2’ or ‘3’ and/or item two to nine = ‘0’or ‘1’ or ‘2’ or ‘3’].

**Mildly food insecure households** [(item one = ‘2’ or ‘3’ or item two = ‘1,’ ‘2,’ or ‘3’ or item three = ‘1’ or item four = ‘1’) and item five to nine = ‘0’].

**Moderately food insecure** [(item three = ‘2’ or ‘3’ or item four = ‘2’ or ‘3’ or item five = ‘1’ or ‘2’ or item six = ‘1’ or ‘2’) and item seven to nine = ‘0’] and

**Severely food insecure households** score [item five = ‘3’ or item six = ‘3’ or item seven = ‘1,’ ‘2,’ or ‘3’ or item eight = ‘1,’ ‘2,’ or ‘3’ or item nine = ‘1,’ ‘2,’or ‘3’] [23] (Table 1).

When there is a response of yes, there is occurrence frequency measurement with three options, ‘Rarely = happened once or twice’, ‘Sometimes = happened three to ten times’ and ‘Often = happened more than ten times’ during the month previous to the survey [23].

**Operational definitions**

**Urban Productive Safety Net Program (UPSNP):** A social protection system which is implementing in the major urban centers of Ethiopia targeting chronic food insecure households and providing cash or food for beneficiaries(households) either “for work”, “for free” or “a combination of both” on a regular basis for five years period [24].

**HFIAS:** Household Food Insecurity Access Scale is a set of questions related to experience of food access that appeared to distinguish food secure from food insecure households across the different culture context, and used to estimate the prevalence of household food insecurity and its category [25].

**Food insecurity:** Food insecurity is state exists when all people do not have adequate physical, social or economic access to sufficient, safe and nutritious food that meets the dietary needs and food preference for an active and healthy life [26].

**Recall period**: The respondent was asked an occurrence question about whether the condition had happened in the past four weeks (30 days) [23].

**Dependency ratio**: It was computed from the ratio of dependent members (age group less than 18 and greater than 64 years) to productive household members (age group 18-64 years) and finally it was grouped in three (tertiles); low, medium and high.

**Household income**: It was computed into tertiles (three group) as low (375-1500 birr), medium (1560-2000 birr) and high (2100-4500 birr) from scores of average household monthly income from any sources.

## Data Collection Tool and Procedure

A pre-tested structured questionnaire was used to collect data on socio-demographic variables and household food insecurity. The socio-demographic part was adapted from published literature and the Ethiopian Demographic and Health Survey [27]. A household food insecurity HFIAS questionnaire was adopted from the FANTA, a validated tool for developing countries food insecurity study, including Ethiopia [23]. The questionnaire was first prepared in English and then translated to Amharic and back again to English by language experts for consistency. Data collectors were 4 health professionals (BSc nurses and public health officers); 2 supervisors had a Master of Public Health (MPH) in Nutrition.

The study participants were mothers from whom data were collected by face-to-face interviews. If mothers were not available at the time of data collection and/or there was no mother at all in the selected household, the head of household was the primary study participant as was recommended by the validated tool developed by FANTA [23]. Daily supervision of the data collection was performed and correction measures were taken daily; if a questionnaire was incomplete the house was re-visited.

## Data Quality Assurance

The quality of the data was ensured through adequate training (one full day) for 4 health professional data collectors (BSc nurses and public health officers) and 2 supervisors (MPH in Nutrition) on the objective of the study, the parts the of questionnaire to assess household food insecurity, the applicability of ethical issues, approaches to interviewing and recording of data. A pre-test was conducted on 5% of the sample size (32 households) from out of the selected study areas in district 05 of Arada sub city, to check the clarity and consistency of the questionnaire before the actual data collection. Amendment of the questionnaires was done based on the findings of the pre-test.

Data were collected by trained health professionals by face-to-face interviewing of mother or head of the household and supervised by trained postgraduate health professional. The completeness of questionnaires was checked daily before data entry. Entered data entry was re-entered on 10% of the sample to check the consistency or reduce data entry error. The internal consistency of the occurrence question (outcome variable measurements) was checked and Cronbach's alpha was found to be 0.897. After the data was entered and cleaned, quality assurance measures were employed using descriptive statistics result from cross-tabulation and frequency distribution before performing statistical analysis. Data were checked for any missing, outliers, category pattern with chi-square assumption.

## Data Management and Statistical Analysis

The completeness of data was checked manually and after editing and clearing, data entry was done by using EpiData version 3.1. After checking the consistency and preparing for analysis, it was export to statistical package for social science (SPSS) version 25. Frequency distribution tables, categorization for continuous and re-categorization for categorical variables using information from different kinds of literature were carried out. Prevalence of household food insecurity and level of food insecurity status (mild, moderate and severe) were computed from the HFIAS nine items of questions (Table 1). The prevalence of food insecurity was calculated by dividing the food insecure households by the total study participant households and then by multiplying 100.

A bi-variable and multivariable logistic regression model was applied with a 95% confident interval [CI]. From the bi-variable logistic regression analysis, variables with *p*<0.25 level of significant also undergo multi-variable logistic regression analysis to determine the independent effect on household food insecurity. The independent variables were entered into multiple analyses through a backward procedure. By adjusting the confounders, variable at *p*-value<0.05 and 95% CI was declared significantly associated with household food insecurity. Multicollinearity was checked among independent variables using standard error; when the value of standard error was more than 2 indicated multicollinearity (maximum SE=0.56). Model fitness was also checked using the Hosmer Lemeshow test with *p*-value >0.05 (*p* = 0.158).

**Ethics approval and consent to participate**

Ethical clearance was obtained from the ethical review board of Saint Paul’s Hospital Millennium Medical College, Department of Public Health. Before the data collection started, an official request for permission to proceed was granted by the Addis Ababa City UPSNP administration office. Before each interview, data collectors explained the purpose of the study, gave assurance of confidentiality, addressed any other ethical issues and obtained informed verbal consent from each household respondent (mother/ head of household).

**Results**

**Socio-demographic Characteristics of the Study Participants**

Of the 624 participants, 607 participated for this study (97.3% response rate). Nearly two-thirds 382(62. 9%) households were headed by a female. One fourth 154 (25.4%) household heads did not have education opportunity (illiterate), whereas 259 (42.7%) attended a secondary level of education. The average family size of the safety net beneficiary households was four. A family size from 1-3 persons was 245 (40.4%). The high dependency ratio among the study participant was 83 (13.7%), whereas the low dependency ratio was 238 (39.2%). About 286 (47.1%) households were found in the middle dependency ratio group. Just more than half 317 (52.2%) of the study participants were married during the survey (Table 2).

**Socioeconomic Characteristics of Study Participants**

Almost one-third 179 (29.5%) of household heads were daily labourers. Three hundred seventy-nine (62.4%) households’ only source of income was from the UPSNP safety net. The mean household monthly income was 65.13±1.18 USD (United States Dollars). The proportion of household income spent on food and food-related items was more than 75% for 431(71%) of households. Two hundred sixty-five (43.7%) of households were found to have a low household income. Nearly one-fourth of the households 143 (23.6%) had at least one individual who suffered from one or more chronic medical conditions (Table 3).

**The occurrence of HFIAS conditions**

The proportion of households experiencing worry about not having enough food was 528 (87%). The majority of the households 456 (75%) replied affirmatively to being unable to eat their preferred food in the 4 weeks before the interview due to a lack of resources (Fig. 1). Households that ate a limited variety of foods in the 4 weeks before the interview due to lack of resources were 462 (76.1%). Households that smaller amounts at a meal or fewer meals than preferred in the 4 weeks before the interview were 426 (70.2%) and 383 (63.1%), respectively. Moreover, households with an affirmative response to the severe conditions of going to sleep hungry or going a whole day and night without food were 219 (36.1%) and 39 (6.4%), respectively (Table 4).

Figure 1. Occurrence of Household Food Insecurity Access Scale conditions among Urban Productive Safety Net Program beneficiary households in Addis Ababa city in June 2019

**The occurrence of household food insecurity domain**

According to their similar characteristics, the nine occurrence questions were grouped into three domains. The result shows that the proportion of household falling in the anxiety domain was 528 (87%), insufficient quality of food domain 463 (76.3%) and insufficient quantity of food and physical consequences domain 440 (72.5%) (Fig 2).

Figure 2. Household food insecurity access scale domain distribution among Urban Productive Safety Net Program beneficiary households in Addis Ababa city June 2019.

**Prevalence of Household food insecurity (HFI)**

The overall prevalence of household food insecurity was 77.1% (468) with (95% CI፡ 73.8-80.7). Four hundred sixty-eight (77.1%) households experienced some extent of food insecurity (mildly food insecure 4 [0.7%], moderately food insecure 129 [21.3%] and severely food insecure 335 [55.2%]) in the 4 weeks before the survey, while 139 (22.9%) households were food secure (Fig 3).

Figure 3. Household food insecurity status among Urban Productive Safety Net Program beneficiary households in Addis Ababa city, June 2019

**Factors associated with household food insecurity**

Based on the result of multivariable analysis, households headed by an uneducated person was 2.56 times [AOR: 2.56; 95%CI: 1.08-6.07] more likely to be food insecure than households headed by individuals who had an education above secondary level, and the odds of food insecurity among households headed by a person with completed secondary-level education were 3.22 times greater [AOR: 3.22; 95%CI: 1.54-6.75] than among households headed by someone who had completed above a secondary level of education (Table 5).

Households with 4 or more family members were nearly 2 times [AOR: 1.87; 95%CI: 1.08-3.23] as likely to suffer from food insecurity as households having less than 4 family members. Likewise, households with a high dependency ratio were almost 4 times [AOR: 3.95; 95%CI: 1.31-11.90] as food insecure as households with a low dependency ratio. Households with no access to credit services were nearly three times [AOR: 2.85; 95%CI:1.25-6.49] more likely to develop food insecurity than households that had access to credit services (Table 5).

The odds of food insecurity among those with a low household income were nearly 5 times [AOR: 4.72; 95%CI: 2.32-9.60] higher than those with high household income; moreover households e with a medium income were 10 times [AOR: 9.78; 95%CI: 4.29-22.35] more likely to experience food insecurity than household with a high income level (Table 5).

**Discussion**

A HFIAS method was carried out using a cross-sectional study to assess the prevalence of household food insecurity among UPSNP beneficiary households in Addis Ababa and multivariable logistic regression analysis was used to identify factors significantly associated with food insecurity. We found that the prevalence of food insecurity was 77.1% and factors significantly associated with food insecurity were education level, dependent ratio, access to credit service and household income.

The prevalence of food insecurity (77.1%) in this study was consistent with a previous study in Addis Ababa (75.0%) [28], East Badawacho District, South Ethiopia (75.8%) [29] and Sekela District, Western Ethiopia (74.1%) [30], but higher than found in several other studies in Addis Ababa (58.16%) [22], Wolaita Soda (37.6%) [31], West and East Gojjam (55.3%) [32]. The higher prevalence in our study might be attributable to our study being conducted among poor households who were UPSNP beneficiaries, whereas the households in other studies were from different parts of Ethiopia which are not safety net beneficiaries. In Addis Ababa, to be eligible for the UPSNP safety net beneficiary program, being poor is a key selection criteria. The variation of these results indicate that area-specific food insecurity surveys are very important to visualize the differences of people’s real situations.

Furthermore, the higher degree of household food insecurity found in this study relative to a national prevalence of food insecurity survey (35.0%) [33] might be due to our very small study area compared to the large coverage of the Ethiopian studies, which allows our findings to give a better insight about food insecurity status in the studied areas.

Our study revealed that 70.2% of households ate smaller amounts at meals than preferred and 63.1% of households also missed some meals during the studied period and 36.1% of the household also reported they went to bed hungry during the survey period. This result was higher than that of study conducted in East Badawacho South Ethiopia [29] that found 62.3% and 10.7% of households were eating a smaller amounts of food and going to sleep at night hungry, respectively. The increment of households experiencing food insecurity conditions might be due to householders’ low purchasing power to access the available food and also the high inflation of food prices at the time of this survey, forcing householders to reduce the variety and amount of food they consumed, especially low-income urban households who spent a large proportion of their income to purchase food.

Our findings of the households falling in anxiety and uncertainties domain (87.0%), insufficient food quantity domain (76.3%), and insufficient food intake and its physical consequence domain (72.5%) were higher than the findings in Wolaita Sodo Town [31] that reported the percentage of households falling in these domains were 37.3%, 37.6% and 33.3%, respectively; and in West Abaya District 38.1%, 38.1%, and 34.5%, respectively [34]. This discrepancy may be attributed to the possibility that vulnerability to food insecurity among the urban poor is higher than among semi-urban and rural households. It can be also explained by the difference that semi-urban and rural households can access food from their garden production; in contrast, urban poor households are dependent on purchasing from market food supply.

This study’s findings that a household head’s educational status of none or primary school completed were significantly associated factor of household food insecurity are consistent with other studies [11, 34, 35]. This might be because when the level of education increases, there is access to better job opportunities and boosting of labour productivity that ultimately increase household income and provide an asset to safeguard the access and utilization of food.

In this study, having a large number of household members increases the likelihood of food insecurity, a finding similar to that of studies conducted in Addis Ababa, South Africa, and Latin America [2, 7, 22, 36]. In contrast with our study, a study in Kenya and another rural parts of north Ethiopia found that households with a large family size were more like to be food secure as compared to those with a small family size [37, 38]. This discrepancy may be due to the fact that as the household size becomes larger, the number of economically active members may also increase; and the increased participation in income-generating opportunities leads to more food security than for households with lower family size.

Households with a high dependency ratio have more chance of being food insecure than those of low dependency ratio groups. This might be because the dependent members are less likely to participate in productive work and their full dependence on others to purchase food and other commodities increases the burden on the household. Our findings were similar to those of several studies in Africa [7, 31, 37].

We also found that a lack access to credit services was one of the predictors of food insecurity as other studies in Ethiopia agreed [22, 35]. This may be due to households that have access to credit participating in diversified income-generating opportunities that are able to boost the financial power of the household. Access to credit services also helps households to cope with the food shortage situation by stabilizing the food purchasing powers of the household. We also found that a household in low and medium income categories were more likely to experience food insecurity than those who had high household income, which is consistent with previous studies conducted in globally, including a study in Ethiopia [2, 7, 11, 31]. The low household income can induce food insecurity; as a vicious cycle, food insecurity also reduces household productivity.

**Limitation of the Study and future research directions**

The estimation of prevalence of household food insecurity relied on a one-month recall of occurrence; recall and social desirability bias could increase or decrease its reported prevalence. However, to overcome such limitations, we used HFIAS validated, highly reliable and culturally sound tool that was able to minimize bias and reveal a relatively good estimate of the prevalence. An attempt also was made to minimize the possibility of bias by explaining the main objectives of the study and by applying appropriate data collection procedures and interview techniques through use of trained data collectors and supervisors. Therefore, generalization of our findings could not be compromised.

In future, the question of whether the UPSNP safety net program creates an attitude of dependency on aid will be useful to investigate in order to provide possible help designing mechanisms to end the need for beneficiaries to receive the aid for so long. Future researchers are also encouraged to investigate the consequences of the UPSNP urban safety net program such as conflict between beneficiaries and non-beneficiaries, food price inflation, extreme weather (drought) capability, and internal population movements and more. It is also recommended to conduct a study on the cause and effect relationship of the UPSNP and poverty to ensure that urban food security status addressed at the country level through time.

**Implications of the study for practice and/or policy**

The rural safety net program is an older project in Ethiopia, whereas the urban safety net program UPSNP is a new program scaled up from the rural experience and started as a pilot in 2017 in urban Ethiopia. One of the strengths of this study was that it investigated food insecurity status of beneficiary households within the pilot project area in Addis Ababa. The findings will have practical application in helping to design a sustainable urban safety net program that improves the livelihoods of current beneficiaries and others in the future. To make a change on the level of food insecurity, this study shows that implementers should give due attention to variables such as household income levels, number of dependent members of beneficiary households, and household access to credit.

Furthermore, the evidence in this study may help programmers and policy makers to give priority to solving the challenges of or predictors for household food insecurity it revealed. Considering the prevalence of food insecurity and associated factors among UPSNP beneficiary households in Addis Ababa City may help the city’s UPSNP agency in collaboration with governmental and non-governmental organization to further scale up programs that will help households help themselves in a sustainable manner. Further, the findings regarding beneficiaries of this urban safety net program may guide its expansion to other urban areas of Ethiopia, thereby reducing national food insecurity levels, and making progress toward the UN Sustainable Development Goal to end all forms of malnutrition.

**Conclusions**

This study showed that three in four households were experiencing food insecurity and that an appropriate multi-dimensional approach including such interventions as improving educational status through formal and vocational training and creating employment opportunities or the opening of small business enterprises through credit service support may help to diversify income sources and to reduce the dependency ratio. The UPSNA should do an impact evaluation of the current safety net program and consider increasing the amount of the per person monthly payment in order to increase family income, which in turn would increase the purchasing power of urban poor households. It is also highly recommended that the urban safety net program give special focus on the creation of sustainable income opportunities for users to improve their means of self-support.

**Abbreviations**

AOR, Adjusted odds ratio; COR, Crude odds ratio; FANTA, Food and Nutrition Technical scale; HFIAS, Household food insecurity access scale; UPSNP, Urban productive safety net program.

**Acknowledgment**

We acknowledge Addis Ababa City Urban Productive Safety Net Program Agency for giving us relevant information for this study. We also highly appreciate the study area’s district administrators for their support by providing necessary information. We thank data collectors, supervisors and household members for their voluntary participation in the study.

**References**

1.	Chowdhury MRK, Khan M, Rafiqul Islam M, Perera NK, Shumack MK, Kader M. Low maternal education and socio‐economic status were associated with household food insecurity in children under five with diarrhoea in Bangladesh. Acta Paediatr. 2016;105(5):555-61.

2.	Smith MD, Kassa W, Winters P. Assessing food insecurity in Latin America and the Caribbean using FAO’s food insecurity experience scale. Food policy. 2017;71(2017):48-61.

3.	FAO I, WFP W, UNICEF. The state of food security and nutrition in the world 2019: Safeguarding against economic slowdowns and downturns. Rome. Food and Agri Org. 2019.

4.	UNICEF. WHO/The World Bank joint child malnutrition estimates-levels and trends. World Health Organization, Geneva The World Bank, Washington, DC. 2012.

5.	Development Initiatives 2018. Global nutrition report: Shining a light to spur action on nutrition. Bristol, UK: Development Initiatives. 2018.

6.	World Bank Group. Poverty and shared prosperity 2018: Piecing together the poverty puzzle. Overview booklet. World Bank, Washington, DC: World Bank Group 2018.

7.	Akinloye O, Putuma M, Adeyefa S. Determinants of food insecurity among the urban poor in the City of Tshwane , South Africa. J Econ Dev Stud. 2016;4(2):101-14.

8.	Eckert S. Urbanization and Health in Developing Countries : A Systematic Review. World Heal Popul 2014;15(1):7-20.

9.	Galal O, Corroon M, Tirado C. Urban environment and health : Food security. Asia-Pacific J Public Heal. 2015;22((3_suppl)):254S-61S.

10.	Global Panel 2017. Urban diets and nutrition: Trends, challenges and opportunities for policy action. Policy brief No. 9. London, UK: Global panel on agriculture and food systems for nutrition. London, UK. 2017.

11.	Moroda G, Tolossa D, Semie N. Food insecurity of rural households in Boset district of Ethiopia : A suite of indicators analysis. Agric Food Secur. 2018;7(65).

12.	Tsegaye AT, Tariku A, Worku AG, Abebe SM, Yitayal M, Awoke T, et al. Reducing amount and frequency of meal as a major coping strategy for food insecurity. Arch Public Health. 2018;76(56).

13.	Mahmudiono T, Nindya T, Andrias D, Megatsari H, Rosenkranz RR. Household food insecurity as a predictor of stunted children and overweight/obese mothers ( SCOWT ). Nutrients. 2018;10(535).

14.	Gebreyesus SH, Endris BS, Hanlon C, Lindtjørn B. Maternal depression symptoms are highly prevalent among food-insecure households in Ethiopia. Public Health Nut 2018;21(5):849-56.

15.	Development Initiatives 2017. Global Nutrition Report 2017: Nourishing the SDGs.Bristol, UK: Development Initiatives. 2017.

16.	The Economist Intelligence Unit 2018. The global food security index 2018: Building resilience in the face of rising food-security risks, the seventh edition of an Economist Intelligence Unit study.The Economist Intelligence Unit. Londone, UK. 2018.

17.	Africa Union. The cost of hunger in Africa : Social and economic impact of child undernutrition in Egypt , Ethiopia , Swaziland and Uganda background paper. Abuja, Nigeria. 2014.

18.	CSA Addis Ababa Ethiopia IRMU. Ethiopia Demographic and Health Survey 2016: Key Indicators Report. 2016.

19.	FDRE. Building an Integrated Safety Net System for the Most Vulnerable Women and Children in Rural and Urban Ethiopia. Addis Ababa, Ethiopia. 2016.

20.	CSA. Central Statistical Authority. 2007 Population and Housing Census of Administrative, Addis Ababa, Ethiopia. 2012.

21.	FDRE. Food and nutrition policy 2018. Federal Democratic Republic of Ethiopia (FDRE). Addis Ababa 2018.

22.	Gebre G. Determinants of food insecurity among households in Addis Ababa City, Ethiopia. Interdiscip Descr Complex Syst. 2012;10(2):159-73.

23.	Coates J, Swindale A, Bilinsky P. Household food insecurity access scale (HFIAS) for measurement of food access: Indicator guide: Version 3. Washington, D.C. Food Nutr Tech Assist Proj Acad Educ. 2007;3:1-32.

24.	Devereux S, Sabates-Wheeler R, Tefera M, Taye H. Ethiopia’s Productive Safety Net Programme (PSNP): Trends in PSNP transfers within targeted households. Sussex, UK and Addis Ababa, Ethiopia: Institute of Development Studies and Indak International Pvt LC. 2006.

25.	Deitchler M, Ballard T, Swindale A, Coates J. Validation of a measure of household hunger for cross-cultural use: Food and Nutrition Technical Assistance II Project (FANTA-2). Washington, DC: FANTA-2. 2010.

26.	Napoli M, De Muro P, Mazziotta M. Towards a Food Insecurity Multidimensional Index. Unpublished Master Thesis Universita Deglistudi Available at< <http://www> fao org/fileadmin/templates/ERP/uni/FIMIPDF. 2011.

27.	CSA, ICF-International. Ethiopia Demographic and Health Survey 2016. Addis Ababa, Ethiopia, and Calverton, Maryland, USA: Central Statistical Agency [Ethiopia] and ORC Macro2016.

28.	Birhane T, Shiferaw S, Hagos S, Mohindra K. Urban food insecurity in the context of high food prices : A community based cross sectional study in Addis Ababa , Ethiopia. BMC Public Health. 2014;14(680).

29.	Betebo B, Ejajo T, Alemseged F, Massa D. Household Food Insecurity and Its Association with Nutritional Status of Children 6 – 59 Months of Age in East Badawacho District , South Ethiopia. J Environ Public Health. 2017;2017(ID 6373595):1-17.

30.	Mulu E, Mengistie B. Household food insecurity and its association with nutritional status of under five children in Sekela District , Western Ethiopia : A comparative cross-sectional study. BMC Nutr. 2017;3(35).

31.	Tantu A, Gamebo T, Sheno B. Household food insecurity and associated factors among households in Wolaita Sodo town , 2015. Agric Food Secur. 2017;6(19).

32.	Motbainor A, Worku A, Kumie A. Level and determinants of food insecurity in East and West Gojjam zones of Amhara Region, Ethiopia: A community based comparative cross-sectional study. . 2016;16(1):1–13. . BMC Public Health. 2016;16(503).

33.	Ethiopian Health and Nutrition Research Institute. Nutrition baseline survey report for the national nutrition program of Ethiopia, Addis Ababa, Ethiopia. 2010.

34.	Shone M, Demissie T, Yohannes B, Yohannis M. Household food insecurity and associated factors in West Abaya district, Southern Ethiopia, 2015. Agric Food Secur. 2017;6(2).

35.	Hassen K, Zinab B, Belachew T. Gender and education as predictors of food insecurity among coffee farming households of the Jimma zone , Southwest of Ethiopia. BMC Nutr. 2016;2(75).

36.	Ndobo F. Determining the food security status of households in a South Afican township. Theses, Boloka Institutional Repository. 2013:1-146.

37.	Scho L, Wekesah F, Mohamed S. Vulnerability to food insecurity in urban slums : Experiences from Nairobi , Kenya. J Urban Heal. 2014;91(6):1098-113.

38.	Alemseged G, Hailu B, ZNZ. Determinants of rural household food insecurity in Northern Ethiopia: An empirical study. Sustain Dev Africa. 2018;20(1):32-63.

**Table 1. Question items 1 to 9 to measure the outcome variable food insecurity**

| **Household Food Insecurity Access Scale question item** | **Yes** | | | **No** |
| --- | --- | --- | --- | --- |
| Worried about not enough food | Rarely [1] | Sometimes [2] | Often [3] | No [0] |
| Unable to eat preferred food | Rarely [1] | Sometimes [2] | Often [3] | No [0] |
| Ate just a few kinds of food | Rarely [1] | Sometimes [2] | Often [3] | No [0] |
| Ate unwanted foods | Rarely [1] | Sometimes [2] | Often [3] | No [0] |
| Ate a smaller than desired amount at a meal | Rarely [1] | Sometimes [2] | Often [3] | No [0] |
| Ate fewer meals in a day than desired | Rarely [1] | Sometimes [2] | Often [3] | No [0] |
| Had no food of any kind | Rarely [1] | Sometimes [2] | Often [3] | No [0] |
| Went to sleep at night hungry | Rarely [1] | Sometimes [2] | Often [3] | No [0] |
| Went without food over a day & night | Rarely[1] | Sometimes [2] | Often [3] | No [0] |

Source: [23].

**Table 2. Socio-demographic characteristics of Urban Productive Safety Net Program beneficiary households in Addis Ababa**

| **Variable** | **Category** | **Frequency (n)** | **Percentage (%)** |
| --- | --- | --- | --- |
| **Sex** | Female | 382 | 62.9 |
|  | Male | 225 | 37.1 |
| **Age (years)** | <40 | 260 | 42.8 |
|  | 40-60 | 268 | 44.2 |
|  | >60 | 79 | 13.0 |
| **Marital status** | Unmarried | 62 | 10.2 |
|  | Widowed | 107 | 17.6 |
|  | Divorced | 121 | 19.9 |
|  | Married | 317 | 52.2 |
| **Educational status** | No education | 154 | 25.4 |
|  | Primary level | 259 | 42.7 |
|  | Secondary level | 107 | 17.6 |
|  | Above secondary | 87 | 14.3 |
| **Family size (persons)*** | 1-3 | 245 | 40.4 |
|  | 4 and above | 362 | 59.6 |
| **Dependency ratio** | Low | 238 | 39.2 |
|  | Middle | 286 | 47.1 |
|  | High | 83 | 13.7 |

*Mean family size (persons) = 4.04±1.75

**Table 3. Socioeconomic characteristics of Urban Productive Safety Net Program beneficiary households in Addis Ababa**

| **Variable** | **Category** | **Frequency (n)** | **Percentage (%)** |
| --- | --- | --- | --- |
| **Source of food** | Prepared at home | 567 | 93.4 |
|  | Bought at restaurant | 31 | 5.1 |
|  | Other | 9 | 1.5 |
| **Gender responsible for food purchasing** | Male | 85 | 14.0 |
|  | Female | 522 | 86.0 |
| **Ownership of the house** | Private rent | 143 | 23.6 |
|  | Government rent | 347 | 57.2 |
|  | Privately owned house | 96 | 15.8 |
|  | Other | 21 | 3.5 |
| **Sources of income** | From UPSNP only | 379 | 62.4 |
|  | From daily labor and UPSNP | 83 | 13.7 |
|  | From pension and UPSNP | 18 | 3.0 |
|  | From self-employment and UPSNP | 127 | 20.9 |
| **Access to credit service** | No | 557 | 91.8 |
|  | Yes | 50 | 8.2 |
| **Access to free health service** | No | 580 | 95.6 |
|  | Yes | 27 | 4.4 |
| **Average monthly household income** | Low | 265 | 43.7 |
|  | Medium | 154 | 25.4 |
|  | High | 188 | 31.0 |
| **Proportion of monthly household income spent on food** | <75% | 176 | 29.0 |
|  | ≥75% | 431 | 71.0 |
| **Household member had history of chronic medical problem*** | Yes | 143 | 23.6 |
|  | No | 464 | 76.4 |

- *History of chronic medical problems included one or more from diabetes, hypertension, HIV/ADIS, TB (tuberculosis bacillus), mental disorder or other chronic health problems.

**Table 4. Occurrence of HFIAS affirmative condition among UPSNP beneficiary households in Addis Ababa**

| **HFIAS affirmative questions** | **Affirmative response (yes)** | | | |
| --- | --- | --- | --- | --- |
|  | **Rarely** | **Sometimes** | **Often** | **Total** |
|  | n(%) | n(%) | n(%) | n(%) |
| Worried about not enough food | 86(14.2) | 114(18.8) | 328(54) | 528(87.0) |
| Unable to eat preferred food | 19(3.1) | 121(19.9) | 316(52.1) | 456(75.2) |
| Ate just a few kinds of food | 14(2.3) | 92(15.2) | 356(58.6) | 462(76.1) |
| Ate unwanted kinds of food | 51(8.4) | 82(13.5) | 18(3.0) | 151(24.9) |
| Ate smaller amount than desired at the meal | 20(3.3) | 177(29.2) | 229(37.7) | 426(70.2) |
| Ate fewer meals than desired in a day | 50(8.2) | 175(28.8) | 158(26.0) | 383(63.1) |
| Had no food of any kind for a day | 87(14.3) | 47(7.7) | 5(0.8) | 139(22.9) |
| Went to sleep at night hungry | 83(13.7) | 130(21.4) | 6(1.0) | 219(36.1) |
| Went without food for a day & night | 38(6.3) | 1(0.2) | 0(0.0) | 39(6.4) |

**Table 5. Factors significantly associated with household food insecurity from multivariable logistic regression analysis**

| **Variables*** | **AOR (95%CI)** | ***P-*value** |
| --- | --- | --- |
| **Educational status** |  |  |
| No education | 2.56(1.08-6.07) | 0.033 |
| Primary level | 3.22(1.54-6.75) | 0.002 |
| Secondary level | 1.00(0.48-2.09) | 0.988 |
| Above secondary level | 1 |  |
| **Family size (persons)** |  |  |
| 1-3 | 1 |  |
| 4 and above | 1.87(1.08-3.23) | 0.025 |
| **Dependent ratio** |  |  |
| Low | 1 |  |
| Middle | 1.57(0.91-2.71) | 0.106 |
| High | 3.95(1.31-11.90) | 0.015 |
| **Access to credit service** |  |  |
| No | 2.85(1.25-6.49) | 0.013 |
| Yes | 1 |  |
| **Household income** |  |  |
| Low | 4.72(2.32-9.60) | <0.001 |
| Middle | 9.78(4.29-22.35) | <0.001 |
| High | 1 |  |

*Variables from the bivariate analysis that has *P*-value<0.25 were adjusted for multivariable analysis: age of household head; marital status of household head; sex of household head; educational status of household head; household family size; dependent ratio; history of medical problem; main sources of household food; gender of food purchaser; proportion of income spent on food; ownership/rental of house; sources of household income; average monthly household income; access to credit services and access to free health service.

Summary Reviewer Comments

The paper presents useful information on food security. A number of comments address the need to more clearly source and date data presented, especially when using those data to support claims that the food insecurity situation is worsening. The absence of baseline data for food insecurity levels in 2017, when the program began, limits the ability to directly compare the levels of food security reported in the survey results with those before the program began. As such the findings report one month’s observations, and while important, are probably insufficient to serve as a basis for program design or policy advice. If future studies are conducted, having some information on food prices would be helpful. A table that clearly shows the three levels of food insecurity and the number used for the dichotomous variable used in the analysis would be useful. It is possible that some of this is in Table 4 which I could not access.
