# Supplementary material for: Food insecurity status and determinants among Urban Productive Safety Net Program beneficiary households in Addis Ababa, Ethiopia
Source: PLoS One. 2021 Sep 27;16(9):e0256634. doi: 10.1371/journal.pone.0256634 (PMC8476016; doi:10.1371/journal.pone.0256634)
Supplement: Attachment — Submitted filename: Comments to the authors.docx [file pone.0256634.s005.docx]

Comments to the authors

This study is very important in terms of developing policies regarding tackling household food insecurity from developing countries perspective. However, this study has been nicely presented, need some focus on the following points,

1. Introduction is too long to read. Some information are surplus like adult and child malnutrition etc. Please provide precious information to make the introduction short.

2. This study is lacking the findings of previous studies to assess the appropriate gap. Please add the proper theoretical framework.

3. I don’t think the section “operational definition” section is adding much in this study. Better to add the study variable list.

4. The “Methodology” section is too long. Data collection tools and procedure and data quality assurance could be readjusted.

5. “Socio-demographic Characteristics of the Study Participants” and “Socioeconomic Characteristics of Study Participants” do not add much significant information in this study. So, need to reorganize in to one para and highlight only key findings.

6. “The occurrence of household food insecurity domain” this section is unclear. How it measured.

7. Why did not you compare the results between UPSNP safety net beneficiary and other available samples.

8. There are numerous studies in many developing countries regarding this issue. What is the difference between current study and previous study except study population?
